# Exploring Social and Locality Variations of Dog Bites in Scotland Using Administrative Data Sources.

**DOI:** 10.23889/ijpds.v7i3.1810

**Published:** 2022-08-25

**Authors:** Jade Hooper, Paul Lambert, Hannah Buchanan-Smith, Tony Robertson

**Affiliations:** 1 University of Stirling

## Objectives

Previous research has shown that hospital admissions for dog-bites are highest in the most deprived areas across England and Wales. In Scotland, thus far there has been no rigorous empirical investigation into social inequalities in dog-related injuries. This study aims to address this gap through analysis of linked administrative data.

## Approach

The study uses administrative health data from NHS24 calls, A&E and SMR01 records involving dog-related injuries linked to Scotland Census micro-data. Area-based measures of social circumstance are considered through the SIMD, along with an exploration of novel, area-level characteristics including measures of local greenspace, average garden size and dog populations. Individual/household level measures of social circumstance taken from the Scottish Census are used to compare the characteristics of individuals with health records involving dog-related injuries to a random sample of individuals not appearing in the health data sets.

## Results

When looking at individual level records and during modelling at aggregate area level, SIMD was an important factor in all models. Whilst some variation was observed across the different types of health data, the number of records, incident risk ratios and odds ratios were all consistently at least 2-3 higher when comparing the most to least deprived areas. Accounting for dog populations and introducing interaction terms for SIMD decile by dog population increased the main effect of SIMD.

When comparing individual/household level measures of social circumstance taken from the Scottish Census, occupation-based measures such as NS-SEC appeared to be relatively important predictors of risk, alongside ethnicity, household composition and age, with children disproportionately represented.

## Conclusion

Incident rates of dog-related injuries were higher in more deprived areas and circumstances where individuals may be seen as more socially disadvantaged at the individual or household level. Social/legal policies related to dog-bites typically don’t consider social disadvantage in any meaningful way. These results show the importance of doing so.

